# Mining biomarkers for type 2 diabetic nephropathy based on urinary proteomics and metabolomics

**DOI:** 10.3389/fendo.2026.1859999

**Published:** 2026-06-22

**Authors:** Mindong Mi, Tianhuan Xiong, Jiyong Gong, Weijie Sun, Tunguang Xu, Qifeng Jiang, Danqing Zhang, Junge Zhang, Jiancheng Huang, Wei Liang

**Affiliations:** 1 Department of Clinical Laboratory, The First Affiliated Hospital of Ningbo University, Ningbo, China; 2 Third School of Clinical Medicine, Zhejiang Chinese Medical University, Hangzhou, China; 3 Department of Nephrology, The First Affiliated Hospital of Ningbo University, Ningbo, China; 4 Zhejiang Engineering Research Center of Innovative Technologies and Diagnostic and Therapeutic Equipment for Urinary System Diseases, Ningbo, China

**Keywords:** amino acids, diabetic kidney disease, proteins, type 2 diabetes, urinary biomarkers

## Abstract

**Background:**

To evaluate and identify urinary biomarkers for the early diagnosis and staging of diabetic kidney disease (DKD).

**Methods:**

This study enrolled 200 participants, including healthy controls (NDM, n=50) and patients with type 2 diabetes. The diabetic patients were stratified by urinary albumin-to-creatinine ratio (ACR) into the following groups: normoalbuminuria (SDM, n=50), microalbuminuria (MADKD, n=50), and macroalbuminuria (ADKD, n=50). Utilizing an integrated multi-dimensional screening strategy, we systematically evaluated traditional urinary protein markers (urinary retinol−binding protein [URBP], urinary immunoglobulin G [UIgG], urinary transferrin [UTRF], urinary alpha−1−microglobulin [Uα1−MG], and urinary beta−2−microglobulin [Uβ2−MG]), 20 urinary amino acids, and urinary proteins identified via high-throughput mass spectrometry. Candidate proteins were validated by ELISA, and their diagnostic performance was assessed using ROC curve analysis, with ACR serving as the practical clinical reference.

**Results:**

Among the traditional urinary protein markers, UTRF and UIgG demonstrated excellent diagnostic value, with areas under the curve (AUC) of 0.926 and 0.916, respectively. Among the amino acids, PRO showed the best diagnostic performance (AUC = 0.746). However, when all 20 urinary amino acids were combined into a diagnostic model, it exhibited outstanding diagnostic value (AUC = 0.928), outperforming individual amino acids and even traditional protein markers. From the top 50 proteins identified in the proteomic screening, serpin family A member 1 (SERPINA1) was determined to be a key protein. Subsequent ELISA validation and ROC analysis further confirmed that SERPINA1 possesses outstanding diagnostic capability (AUC = 0.964, 95% CI: 0.936–0.990), significantly outperforming other candidate proteins, namely osteoclast−associated Ig−like receptor (OSCAR), contactin 1 (CNTN1), and CD58 molecule (CD58).

**Conclusions:**

Traditional proteins, especially UTRF/UIgG, hold diagnostic value. The 20-amino-acid combination (AUC = 0.928) outperformed them. This study first systematically identifies urinary SERPINA1 as a highly promising DKD biomarker, offering a novel target for early diagnosis and precise management.

## 1 Introduction

Diabetic Kidney Disease (DKD) is one of the most frequent microvascular complications of diabetes and poses a significant global public health burden. This burden is strongly associated with the growing prevalence of diabetes, which is a key driver of DKD incidence. Approximately 30%-40% of diabetic patients develop DKD, which is responsible for 40-50% of all end-stage renal disease (ESRD) cases requiring dialysis (1, 2). With the population of type 2 diabetes patients in China surpassing 140 million, DKD has emerged as the leading cause of chronic kidney disease (CKD). Epidemiological studies demonstrate that the prevalence of diabetic kidney disease (DKD) among diabetic patients in China ranges from 20% to 40%, and its cumulative incidence rises substantially with longer duration of diabetes (3). The high incidence of DKD presents a considerable public health challenge, which in turn necessitates the development of early detection methods.

The current clinical diagnosis of DKD is based on the estimated glomerular filtration rate (eGFR) and the urinary albumin excretion rate (UACR), with the detection of microalbuminuria (30–300 mg/24 h) playing a key role in early diagnosis (4). Nevertheless, this diagnostic strategy is subject to significant limitations. A major drawback is that structural renal injury—manifesting as glomerular basement membrane thickening and mesangial matrix expansion—can occur in 20-40% of DKD patients before the appearance of microalbuminuria (2, 3). Additionally, albuminuria measurement is characterized by high daily variability (coefficient of variation up to 40-100%). This susceptibility to factors such as exercise and blood pressure instability results in substantial rates of false-positive and false-negative diagnoses (3, 4). Furthermore, “non-proteinuric DKD” is observed in 15-45% of individuals with type 2 diabetes. These patients experience a rate of renal function decline comparable to those with proteinuric DKD, yet they remain undetectable by current diagnostic criteria (2). This significant limitation underscores the critical need to discover novel biomarkers with superior sensitivity and specificity, which has accordingly emerged as a major research focus.

The application of proteomics and metabolomics technologies has facilitated the discovery of a series of biomarkers with diagnostic potential and prognostic value. Research confirms that the urinary protein classifier CKD273 (a model containing multiple peptide biomarkers) can effectively predict DN progression. This not only represents a more accurate risk assessment tool but also marks an important step toward personalized nephrology (5).

In recent years, urinary metabolomics studies have provided new perspectives for the early identification of diabetic kidney disease (DKD). Changes in the urinary levels of branched-chain amino acid (BCAA) metabolites and aromatic amino acids have shown significant predictive value (6, 7). Studies have found that elevated urinary 3-hydroxyisobutyrate (a metabolite of valine) (8) is significantly associated with a rapid decline in glomerular filtration rate (eGFR) and an increased risk of end-stage renal disease (ESRD). On the other hand, decreased urinary tyrosine levels have been confirmed to be closely related to the occurrence of proteinuria and DKD progression, reflecting abnormal metabolite excretion due to impaired tubular function (9). Additionally, decreased levels of glycine and histidine are closely associated with inflammation, oxidative stress, and poor prognosis in DKD (10). These urinary amino acid metabolites provide promising biomarkers for the non-invasive early diagnosis and prognostic assessment of DKD.

Advancing urinary biomarker research for DKD requires moving beyond the limitations of single methodologies. The key lies in integrating diagnostic markers discovered through different methodological approaches (such as proteomics and metabolomics) and leveraging their combined application to achieve complementary strengths, thereby constructing more robust and accurate diagnostic models. This study established a novel “tripartite” multi-dimensional biomarker screening system, designed to systematically discover new biomarker clusters with high diagnostic value. This system integrates three dimensions: conventional renal injury markers in urine, such as α1-microglobulin and β2-microglobulin; urinary amino acid profiling that reflects DKD-specific metabolic disturbances; and the in-depth mining of urinary proteins using high-resolution mass spectrometry. By leveraging the complementarity of multi-dimensional information, this system is expected to significantly enhance the diagnostic performance for DKD.

## 2 Materials and methods

### 2.1 General data

A total of 150 patients with type 2 diabetes mellitus (T2DM) were recruited from the outpatient and inpatient departments of Ningbo University Affiliated First Hospital between June and December 2024. Based on the urinary albumin-to-creatinine ratio (ACR), the patients were categorized into three groups: the normoalbuminuria group (SDM, n=50; ACR<30 mg/g), comprising 37 males and 13 females with a mean age of 55.88 ± 12.37 years; the microalbuminuria group (MADKD, n=50; 30mg/g≤ACR<300 mg/g), comprising 36 males and 14 females with a mean age of 55.42 ± 14.29 years; and the macroalbuminuria group (ADKD, n=50; ACR≥300 mg/g), comprising 37 males and 13 females with a mean age of 54.23 ± 13.07 years. Additionally, 50 healthy individuals (NDM, n=50) from the health examination center during the same period served as the control group. This group included 27 males and 23 females with a mean age of 40.77 ± 10.17 years.

### 2.2 Inclusion and exclusion criteria

The inclusion criteria for the T2DM group were: (i) meeting the diagnostic standards of the Chinese Guidelines for the Prevention and Treatment of Type 2 Diabetes (2020 Edition) (11); and (ii) age >18 years. Healthy controls were enrolled if they had normal fasting glucose (<6.1 mmol/L), normal key biochemical indices (including liver and kidney function), and no significant chronic disease history.

Exclusion Criteria: Patients were excluded for: 1) type 1 diabetes; 2) acute diabetic complications within 3 months; 3) concurrent infections, severe consumptive diseases, or hereditary kidney diseases; 4) history of renal replacement therapy; 5) severe hepatic dysfunction; or 6) uncontrolled cardiovascular/cerebrovascular or significant endocrine diseases.

Ethical Approval: The study protocol was reviewed and approved by the Medical Ethics Committee of Ningbo University Affiliated First Hospital (Approval No. 2024-152A; Date: 2025-02-28). Written informed consent was obtained from all participants prior to their enrollment in the study.

### 2.3 Research methods

#### 2.3.1 Traditional urinary protein biomarkers

After an overnight fast of ≥8 hours, fasting blood and random mid-stream urine were collected. Blood was drawn into serum separator and EDTA-K2 tubes. The serum tube was centrifuged (3000 r/min, 5 min) to obtain serum, and the urine sample was centrifuged (1500 r/min, 5 min) to obtain the supernatant. All samples were aliquoted and stored at -80 °C for subsequent analysis.

Serum creatinine (Crea) and glucose (Glu) were assayed enzymatically on a Beckman AU5800 analyzer with reagents from Beckman Coulter (USA), and the eGFR was derived from the CKD-EPI equation. HbA1c was quantified directly from EDTA whole blood via ion-exchange HPLC (Tosoh Corporation) using the manufacturer’s original reagents.

Urinary creatinine (UCrea) was measured enzymatically (Beckman AU5800; Beckman Coulter reagents). Urinary immunoglobulin G (UIgG), retinol-binding protein (URBP), α1-microglobulin (Uα1-MG), β2-microglobulin (Uβ2-MG), and transferrin (UTRF) levels were quantified by immunoturbidimetry (Beckman AU5800; reagents from Bairong Diagnostic Products, Shanghai). To correct for variations in urine concentration, all urinary protein levels are reported as ratios to UCrea.

#### 2.3.2 20 Amino acids

The concentrations of 20 amino acids in urine samples were quantified using liquid chromatography-tandem mass spectrometry (LC-MS/MS). Samples were pretreated with an amino acid extraction kit (Hangzhou puju Medical Technology Co.) according to the manufacturer’s protocol, which included protein precipitation and derivatization steps. Chromatographic separation was performed on a C18 column maintained at 40 °C. Mass spectrometric detection was carried out using electrospray ionization (ESI) in positive ion mode with multiple reaction monitoring (MRM). Quantification was achieved using isotope-labeled amino acids as internal standards, and the concentrations of individual amino acids were calculated based on a calibrated standard curve.

The 20 amino acids are: alanine (ALA), valine (VAL), leucine (LEU), isoleucine (ILE), proline (PRO), phenylalanine (PHE), tryptophan (TRP), methionine (MET), glycine (GLY), tyrosine (TYR), serine (SER), threonine (THR), aspartic acid (ASP), asparagine (ASN), lysine (LYS), arginine (ARG), histidine (HIS), glutamic acid (GLU), citrulline (CIT), and ornithine (ORN).

#### 2.3.3 Proteomics

For proteomic analysis, a pooled sample was created by combining equal aliquots (200μL each) from 10 randomly selected individual urine samples within each group. This process was repeated to prepare three independent biological replicate pools per group.

Following thawing at 4°C, body fluid samples were depleted of high-abundance proteins using a HighSelect™ Top14 kit. The protein solution was brought to 100μL with 50 mM ammonium bicarbonate, denatured (95°C, 3 min), and digested with trypsin (37°C, 16 h) after cooling. The extracted and lyophilized peptides were desalted, reconstituted in 0.1% formic acid, and 2% of the sample was injected for analysis.

Peptide separation was carried out on an EASY-nLC 1200 nano-HPLC system using a 10-cm C18 analytical column (75μm ID, 1.9μm) at a flow rate of 600nL/min, with 0.1% formic acid in water (A) and 0.1% formic acid in 80% acetonitrile (B) as mobile phases, following column equilibration with 100% A.

Instrument: Following chromatographic separation, peptides were analyzed using an Orbitrap HF-X (HFX) mass spectrometer (Thermo Fisher Scientific, Waltham, MA, USA) operated in positive ion mode. Full MS1 scans were acquired over the m/z range of 300–1400 with a resolution of 60,000 (at m/z 200), an automatic gain control (AGC) target of 3e6, and a maximum injection time of 20ms. Data-independent acquisition (DIA) was performed by isolating 30 variable windows per cycle following each full scan. MS2 spectra were generated using higher-energy collisional dissociation (HCD) with a normalized collision energy of 27% and were acquired at a resolution of 15,000 (at m/z 200).

#### 2.3.4 ELISA validation

The levels of four target proteins (SERPINA1, OSCAR, CNTN1, CD58) were quantified by indirect ELISA. Primary antibody details are provided in **Table 1**. Briefly, samples diluted in CBS buffer (0.05M, pH9.6) were coated onto plates (100μL/well) overnight at 4°C. After washing with PBST (0.05% Tween-20), wells were blocked with 1% gelatin in PBS (200μL/well, 37°C, 2h). Primary antibodies diluted in PBST were added (100μL/well, 37°C, 1 h), followed by incubation with an HRP-conjugated goat anti-rabbit secondary antibody (1:4000, 100μL/well, 37°C, 30mins). After TMB substrate incubation (100μL/well, 37°C, 10mins), the reaction was stopped with 2 M H_2_SO_4_, and absorbance was measured at 450 nm. Blank and negative controls were included in each assay, and sample levels were expressed as optical density (OD) values.

**Table 1:** Antibodies and their sample dilution factors.

Antibody	Brand	Catalog no.	Antibody dilution	Sample dilution
SERPINA1	ABclonal	A21972	10,000	50
OSCAR	Proteintech Group, Inc	21996-1-AP	10,000	10
CNTN1	Proteintech Group, Inc	13843-1-AP	4,000	2
CD58	Proteintech Group, Inc	10878-1-AP	4,000	2

### 2.4 Statistical methods

Data were analyzed using SPSS 26.0 and R language (v4.3.1).

#### 2.4.1 Intergroup comparisons

The normality of continuous variables was assessed using the Shapiro-Wilk test. Data with a normal distribution are expressed as the mean ± standard deviation and were compared between groups using one-way analysis of variance (ANOVA). Non-normally distributed data are expressed as the median and interquartile range (IQR), and group comparisons were performed using the Kruskal-Wallis H test. Categorical variables are presented as numbers (percentages) and were analyzed using the Chi-square test or Fisher’s exact test, as appropriate.

#### 2.4.2 Diagnostic efficacy evaluation

Using the urinary albumin-to-creatinine ratio (ACR) as a practical clinical reference for DKD staging, we evaluated the diagnostic value of conventional urinary proteins, urinary amino acids, and candidate urinary protein biomarkers. While ACR-guided stratification is widely accepted for clinical staging, it is not a true histopathological gold standard; nevertheless, it serves as a clinically meaningful anchor for biomarker evaluation in this context. Receiver operating characteristic (ROC) curves were plotted, and the area under the curve (AUC) along with its 95% confidence interval (CI) served as the measure of diagnostic accuracy. The optimal cutoff value was determined by maximizing the Youden’s index (J=sensitivity+specificity-1), with the corresponding sensitivity and specificity reported. Additionally, logistic regression models were constructed to establish the combined diagnostic models for all 20 urinary amino acids and for the five key amino acids, respectively.

#### 2.4.3 Omics data analysis

Proteomics: Differentially expressed proteins were screened with |log_2_FC|≥1.0 and FDR-corrected *P* < 0.05. Trend analysis: Spearman ρ was calculated to assess monotonicity during disease progression (|ρ|≥0.8 for strong trends). Cluster analysis: Unsupervised clustering was performed using the Mfuzz package (FCM algorithm) based on Z-score normalized expression levels. Omics data used the Benjamini-Hochberg method to control FDR<0.05.

## 3 Results

### 3.1 Intergroup difference analysis

#### 3.1.1 Demographic characteristics and baseline indicators

This study enrolled 200 participants, categorized into four groups: the healthy control group (NDM, n=50), the normoalbuminuric diabetes group (SDM, n=50), the microalbuminuric group (MADKD, n=50), and the macroalbuminuric group (ADKD, n=50). As shown in **Table 2**, there was no statistically significant difference in sex distribution among the groups (*P* = 0.108). All diabetic groups were significantly older than the NDM group (all *P* < 0.0001, **Table 3**), while no significant age difference was observed among the diabetic subgroups themselves (*P*>0.05).

**Table 2:** Demographic data and observed indicators of study subjects.

Group	NDM	SDM	MADKD	ADKD	*P*
Number of Cases	Male: 26 (52.0%)	Male: 37 (74.0%)	Male: 35 (70.0%)	Male: 33 (66.0%)	0.108
	Female: 24 (48.0%)	Female: 13 (26.0%)	Female: 15 (30.0%)	Female: 17 (34.0%)	
Age	39.00 [32.00;46.75]	55.88 ± 12.37	55.84 ± 14.15	55.00 ± 12.86	<0.001
Crea	68.36 ± 13.87	76.92 ± 20.04	71.50 [60.25;85.25]	88.000 [70.250;134.000]	<0.001
Glu	4.927 ± 0.472	7.045 [5.898;9.258]	6.790 [6.168;8.133]	7.050 [5.912;8.182]	<0.001
eGFR	108.02 ± 10.43	92.06 ± 19.93	96.50 [78.25;109.00]	74.08 ± 32.25	<0.001
HBA1c	5.60 [5.30;5.60]	7.00 [6.40;8.25]	6.80 [6.13;7.90]	6.85 [6.33;8.00]	<0.001
UCrea	15616.0 ± 6451.1	11620.5 [9073.0;16187.5]	10826.9 ± 3973.5	7754. 5 ± 2467.3	<0.001
ACR	4.967 [4.147;7.146]	12.988 [8.210;17.383]	88.145 [64.519;122.537]	903.658 [516.687;1561.921]	<0.001
UIgG/C	3.587 [2.592;4.960]	6.076 ± 3.963	13.083 [6.361;19.479]	68.035 [44.014;118.466]	<0.001
URBP/C	0.116 ± 0.046	0.280 [0.138;0.510]	0.272 [0.154;0.675]	1.023 [0.382;4.923]	<0.001
Uα1-MG/C	3.091 [2.120;5.531]	13.368 ± 8.145	12.701 [7.128;19.673]	27.136 [15.524;52.819]	<0.001
Uβ2-MG/C	0.083 [0.068;0.120]	0.168 [0.086;0.565]	0.126 [0.051;0.419]	0.791 [0.164;7.228]	<0.001
UTRF/C	0.315 [0.208;0.498]	1.045 [0.308;1.742]	6.092 [2.507;9.776]	50.928 [29.542;85.152]	<0.001

The Shapiro-Wilk test was used for normality testing. Data with a normal distribution (*P*>0.05) are presented as Mean ± Standard Deviation (Mean ± SD). Data with a non-normal distribution (*P ≤* 0.05) are presented as Median [Interquartile Range] (Median [IQR]). The P represents the result of the hypothesis test (Kruskal-Wallis test) for between-group comparisons.

**Table 3:** Pairwise comparison results of demographic data from study subjects.

Group(*P*)	NDM/SDM	NDM/MADKD	NDM/ADKD	SDM/MADKD	SDM/ADKD	MADKD/ADKD
Age	<0.0001	<0.0001	<0.0001	0.988	0.7577	0.7522
Crea	0.2911	0.0831	<0.0001	0.4354	0.0001	0.0007
Glu	<0.0001	<0.0001	<0.0001	0.6758	0.7721	0.8738
eGFR	0.0011	0.0003	<0.0001	0.6518	0.0004	0.0012
HBA1c	<0.0001	<0.0001	<0.0001	0.3211	0.4561	0.7533
UCrea	0.011	<0.0001	<0.0001	0.0385	<0.0001	0.0032
ACR	0.9655	0.5731	<0.0001	0.5752	<0.0001	<0.0001

Renal function indices exhibited changes characteristic of disease progression; however, estimated glomerular filtration rate (eGFR) and serum creatinine (Crea) differed in their sensitivity. eGFR showed a continuous, stepwise decline from the NDM group to the ADKD group (*P* < 0.01). Among the pairwise comparisons, only the difference between the SDM and MADKD groups was not significant (*P* = 0.6518), while all other pairwise comparisons yielded statistically significant differences. In contrast, Crea levels also showed no significant change among the early-stage groups but were markedly elevated in the ADKD group, demonstrating significant differences compared to the NDM, SDM, and MADKD groups (all *P* < 0.01). These findings suggest that eGFR may provide an earlier overall indication of declining renal function, whereas a significant elevation in Crea is more commonly observed in advanced disease stages, reflecting their distinct clinical sensitivities at different phases of diabetic kidney disease.

#### 3.1.2 Glycometabolism and proteinuria markers

Analysis of glycemic parameters revealed that fasting blood glucose (Glu) and glycated hemoglobin (HbA1c) levels were significantly elevated in all diabetic subgroups (SDM, MADKD, ADKD) compared with the healthy control group (NDM) (all *P* < 0.0001). However, no statistically significant differences in Glu or HbA1c were observed in pairwise comparisons among the diabetic subgroups themselves (all *P*>0.05). This pattern likely reflects the effect of clinical glucose-lowering interventions; despite the progression of diabetic kidney disease, glycemic levels were maintained at a relatively consistent range across different albuminuria stages due to pharmacological management, thus showing no further deterioration.

**Figure 1:**
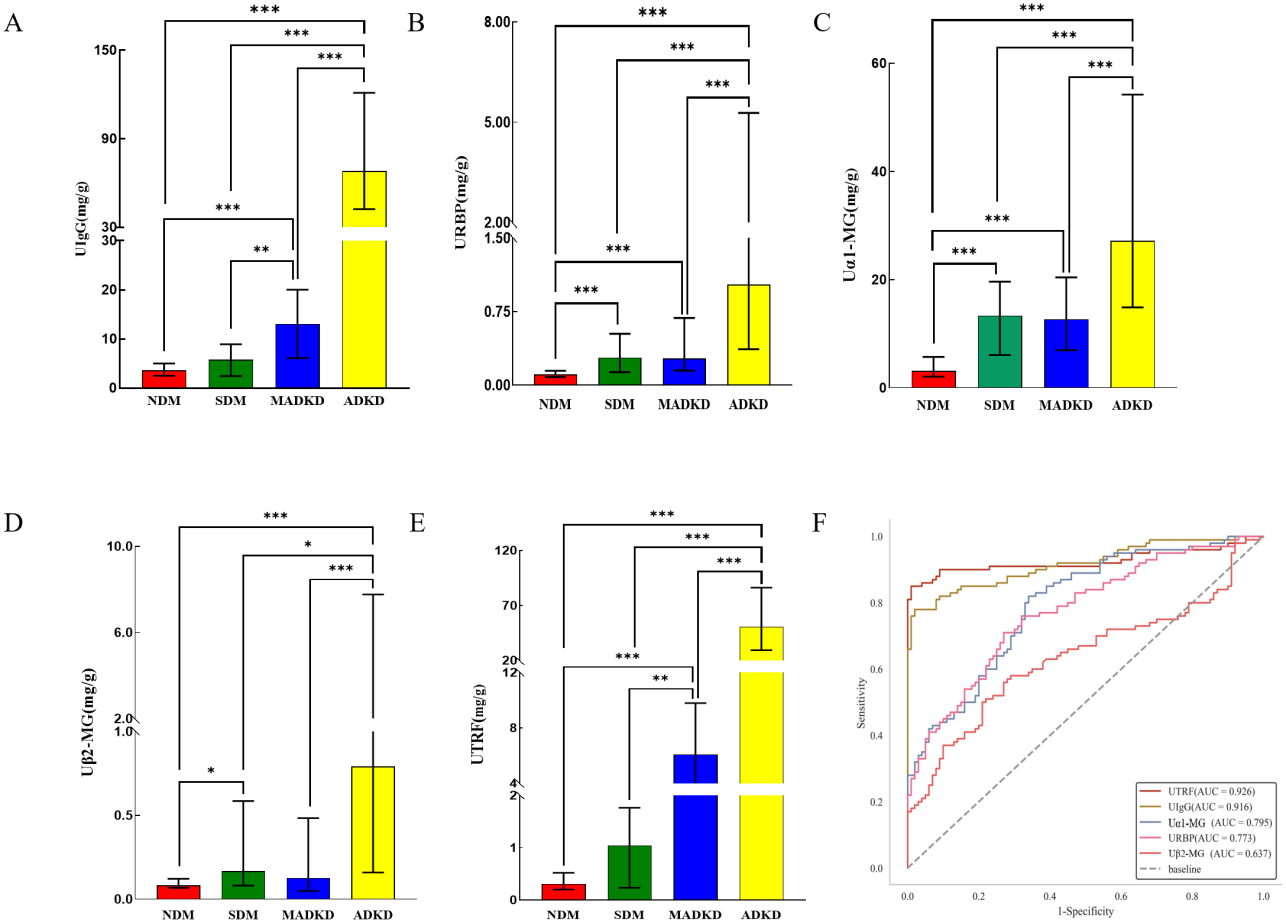
The Diagnostic Performance of Five Traditional Proteins in Type 2 Diabetic Kidney Disease. **(A)** Pairwise Comparison of UIgG. **(B)** Pairwise Comparison of URBP. **(C)** Pairwise Comparison of Ua1-MG. **(D)** Pairwise Comparison of Uβ2-MG. **(E)** Pairwise Comparison of UTRF. **(F)** ROC Curves of the Five Traditional Proteins. *P<0.05, ** *P*<0.01, *** *P*<0.001.

As shown in **Figures 1A–E**, the five target proteins exhibited highly significant differences (all *P* < 0.001) in pairwise comparisons between the ADKD group and the NDM, SDM, and MADKD groups, indicating universal sensitivity of this protein panel in advanced-stage Diabetic Kidney Disease (DKD). Significant differences were observed in UTRF and UIgG between the SDM and MADKD, suggesting that changes in their urinary concentrations may predict the risk of progression from SDM to MADKD in diabetic patients and aid in identifying early renal impairment. URBP and Uα1-MG showed highly significant differences (all *P* < 0.001) in comparisons between the healthy control group (NDM) and all diabetic groups (SDM, MADKD, and ADKD). This pattern indicates that URBP and Uα1-MG may undergo alterations at the very early stages of diabetic kidney injury, even when urinary protein levels remain normal, making them strong candidate biomarkers for early screening.

#### 3.1.3 Amino acid metabolic profile characteristics

**Figure 2:**
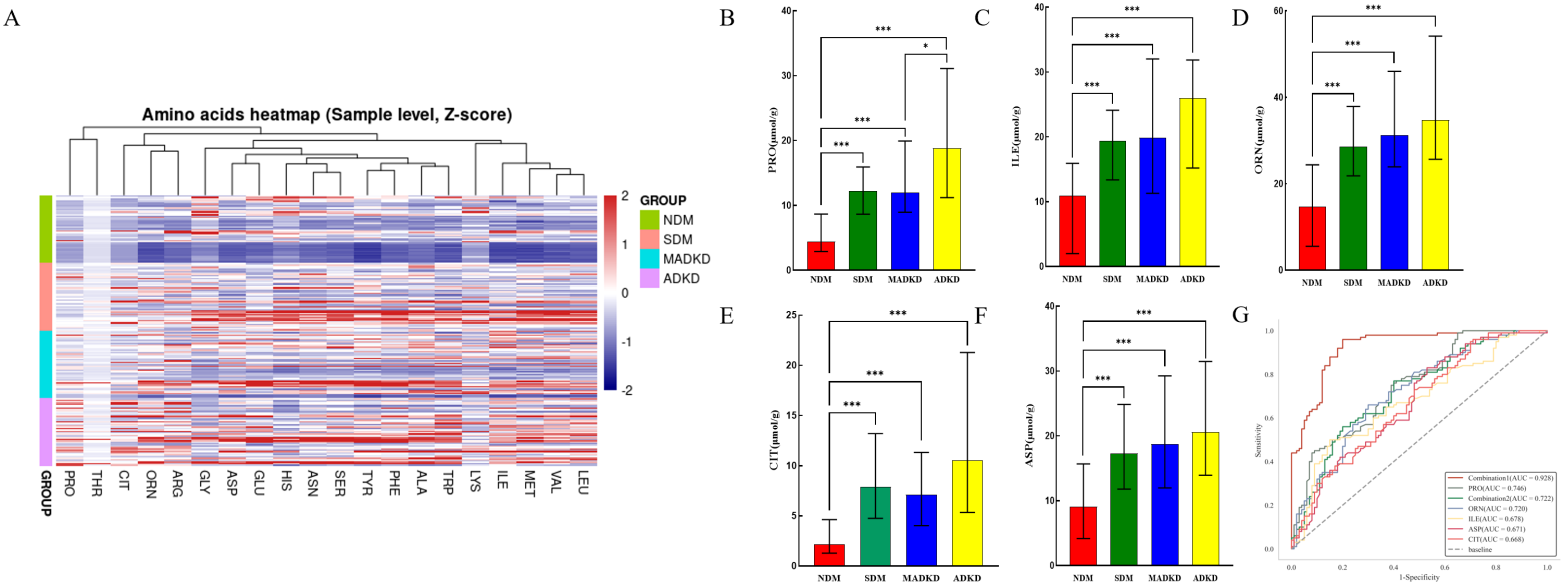
The Diagnostic Performance of Amino Acids in Type 2 Diabetic Kidney Disease. **(A)** Heatmap Analysis of the 20 Amino Acids. **(B)** Pairwise Comparison of PRO. **(C)** Pairwise Comparison of ILE. **(D)** Pairwise Comparison of ORN. **(E)** Pairwise Comparison of CIT. **(F)** Pairwise Comparison of ASP. **(G)** ROC Curves of the Five Key Amino Acids. *P<0.05, ** *P*<0.01, *** *P*<0.001.

Based on the amino acid heatmap shown in **Figure 2A**, the expression levels of the vast majority of amino acids exhibited a stepwise increase with the progression of DKD. This upregulation was particularly pronounced in the ADKD stage, where multiple amino acids were significantly elevated, suggesting a state of systemic metabolic disturbance. The top 10 most differentially abundant urinary amino acids were identified (as shown in **Table 4**). Pairwise comparisons for five key amino acids among these—illustrated in **Figures 2B–F**—revealed that, except for proline (PRO) which showed a significant difference between the ADKD and MADKD groups, none of the others demonstrated statistically significant differences across the diabetic clinical stages (SDM, MADKD, ADKD). In contrast, all five key amino acids exhibited highly significant differences between each diabetic group (SDM, MADKD, ADKD) and the healthy control (NDM) group. These results indicate that while urinary amino acid profiles can effectively distinguish diabetic patients from healthy individuals, their value in differentiating between the various clinical stages of DKD is limited. Notably, the dynamic changes in PRO levels throughout disease progression highlight its potential as a specific biomarker for DKD progression, whereas the other amino acids are more likely to reflect the broader metabolic disturbances associated with diabetes itself.

**Table 4:** Top 10 amino acids with the most significant differences.

Group	NDM	SDM	MADKD	ADKD	*P*
PRO	4.460 [2.933;8.370]	12.186 [8.727;15.875]	12.000 [9.207;19.775]	18.800 [11.525;30.775]	<0.001
ORN	14.650 [5.721;23.725]	28.627 [22.200;37.775]	31.300 [24.700;45.325]	34.750 [26.225;51.473]	<0.001
ILE	10.900 [2.109;15.800]	19.350 [13.650;23.675]	19.900 [11.525;31.846]	26.000 [15.675;31.725]	<0.001
ASP	9.047 [4.319;15.325]	17.300 [11.925;24.329]	18.750 [12.300;28.875]	20.600 [13.955;31.300]	<0.001
CIT	2.200 [1.300;4.572]	7.865 [4.820;12.775]	7.115 [4.203;11.000]	10.525 [5.740;20.275]	<0.001
TRP	26.150 [12.900;43.098]	56.550 [33.225;69.225]	48.800 [31.925;89.925]	57.006 ± 28.707	<0.001
ARG	7.470 [4.135;16.625]	17.000 [12.750;26.650]	17.500 [10.185;27.450]	21.550 [13.519;36.650]	<0.001
VAL	23.800 [10.238;33.178]	39.900 [30.125;68.075]	41.900 [27.150;69.672]	49.800 [34.625;65.775]	<0.001
ALA	87.700 [39.447;125.984]	230.848 [135.500;347.500]	233.000 [104.750;405.750]	230.000 [146.250;353.000]	<0.001
LEU	17.300 [5.435;26.482]	28.850 [22.400;48.316]	27.300 [17.725;49.500]	35.850 [22.678;50.984]	<0.001

The Shapiro-Wilk test was used for normality testing. Data conforming to a normal distribution (*P*>0.05) are expressed as Mean ± SD; data not conforming to a normal distribution (*P ≤* 0.05) are expressed as Median [IQR]. The P-value represents the result of the Kruskal-Wallis test for between-group comparisons. All amino acid results were corrected using urinary creatinine.

### 3.2 Receiver operating characteristic curve analysis

The diagnostic efficacy of traditional urinary protein biomarkers and five key amino acids for diabetic kidney disease was assessed using receiver operating characteristic (ROC) curves with the urinary albumin-to-creatinine ratio (ACR) serving as a practical clinical reference (**Figures 1F, G**; **Table 5**). Among the five traditional urinary protein biomarkers, UTRF and UIgG demonstrated outstanding diagnostic value, with area under the curve (AUC) values reaching 0.926 and 0.916, respectively. At a cut-off value of 3.538mg/g, UTRF achieved 85% sensitivity and 99% specificity (Youden’s index=0.84). UIgG at a cut-off value of 11.617mg/g showed 78% sensitivity and 98% specificity (Youden’s index=0.76). Uα1-MG and URBP exhibited moderate diagnostic performance with AUCs of 0.795 and 0.773, respectively, while Uβ2-MG showed relatively limited diagnostic value (AUC = 0.637).Among the five key urinary amino acids, PRO demonstrated the best diagnostic performance (AUC = 0.746), with 77% sensitivity and 59% specificity at a cut-off value of 9.4μmol/g. ORN (AUC = 0.720) showed a good balance between sensitivity and specificity (66% and 70%, respectively) at a cut-off value of 28.9μmol/g. ILE, ASP, and CIT had similar AUC values, with ILE showing higher specificity (85%) while ASP demonstrated better sensitivity (83%).

**Table 5:** ROC analysis of traditional urinary proteins and five key urinary amino acids.

Feature	AUC	Sensitivity	Specificity	Youden’s index	Cut-off
Traditional Urinary Proteins					
UTRF	0.926	0.85	0.99	0.84	3.538mg/g
UIgG	0.916	0.78	0.98	0.76	11.617mg/g
Uα1-MG	0.795	0.82	0.66	0.48	9.237mg/g
URBP	0.773	0.76	0.68	0.44	0.208mg/g
Uβ2-MG	0.637	0.5	0.79	0.29	0.212mg/g
Five Key Urinary Amino Acids					
Combination 1	0.928	0.96	0.80	0.76	0.341
PRO	0.746	0.77	0.59	0.36	9.4μmol/g
Combination 2	0.740	0.56	0.85	0.41	0.571
ORN	0.720	0.66	0.7	0.36	28.9μmol/g
ILE	0.678	0.5	0.85	0.35	24.0μmol/g
ASP	0.671	0.83	0.47	0.3	12.0μmol/g
CIT	0.668	0.73	0.52	0.25	4.79μmol/g

Additionally, ROC curves were constructed for the combined diagnostic model of all 20 amino acids (Combination 1) and the combined diagnostic model of the five key amino acids (Combination 2). The combined model of the 20 amino acids demonstrated outstanding diagnostic value (AUC = 0.928), whereas the combined model of the five key amino acids showed only moderate diagnostic value (AUC = 0.740).

Based on the comprehensive results, the diagnostic performance of the two types of biomarkers can be ranked as follows: Combination 1>UTRF>UIgG>Uα1−MG>URBP>PRO>Combination 2>ORN>ILE>ASP>CIT>Uβ2−MG. Traditional urinary protein biomarkers significantly outperformed the key urinary amino acids in overall diagnostic performance. Notably, the performance of individual markers revealed that urinary amino acids had lower diagnostic efficiency than traditional urinary proteins; however, when all 20 amino acids were combined for diagnosis, the combined model (Combination 1) exhibited the best diagnostic performance.

### 3.3 Proteomics

#### 3.3.1 Mass spectrometry data quality control

**Figure 3:**
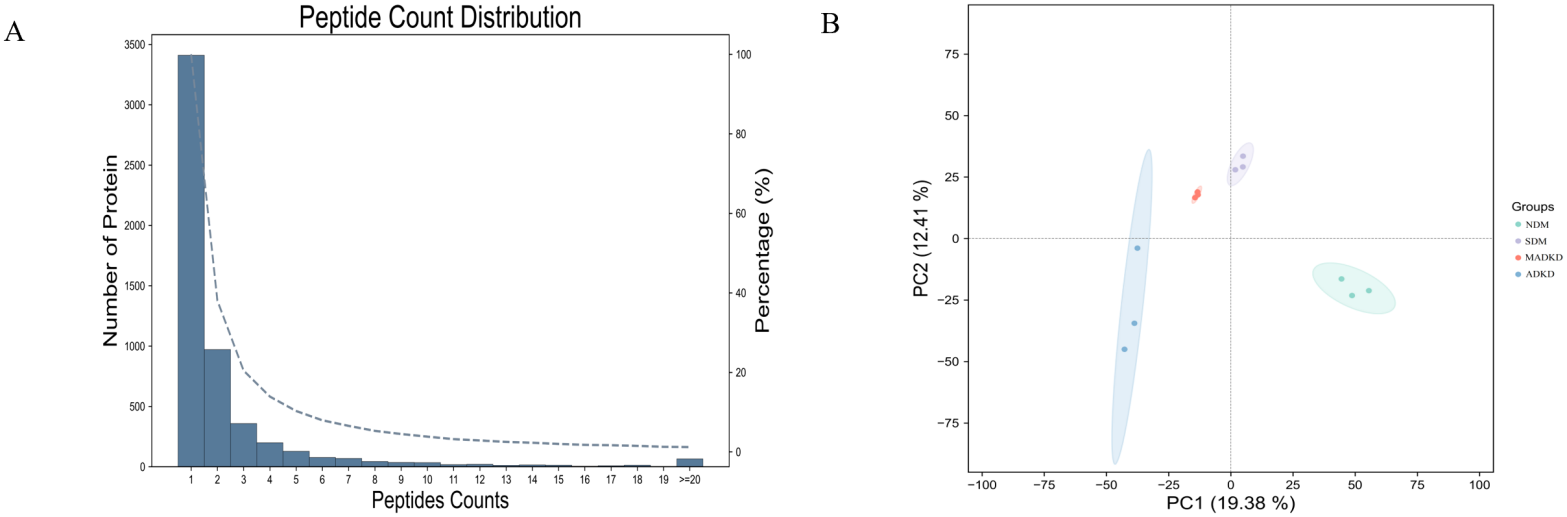
Proteomics MS Data Quality Control. **(A)** Peptide Count Distribution per Protein. **(B)** PCA Score Plot.

This experiment was performed using a high-mass-accuracy and high-resolution liquid chromatography-tandem mass spectrometry (LC-MS/MS) system, which maintained minimal mass deviation during data acquisition and ultimately produced high-quality MS1 and MS2 spectra. The mass errors for the vast majority of identified peptides were confined within 10 ppm, confirming the accuracy and reliability of the identifications. The spectra were then analyzed using the Mascot search engine, which assigned a confident score to each MS2 spectrum. The excellent distribution of peptide identification scores, as shown in **Figure 3A**, further demonstrates the ability of the instrument to generate high-quality experimental data. Moreover, a stringent peptide false discovery rate (FDR) threshold of ≤0.05 was applied as the filtering criterion for all qualitative analyses of the data-independent acquisition (DIA) data.

To investigate variations in the major urinary components among the four groups, principal component analysis (PCA) was performed on all samples. The results revealed a distinct separation among the four urine sample groups in both positive and negative ion modes (**Figure 3B**).

#### 3.3.2 Difference analysis

**Figure 4:**
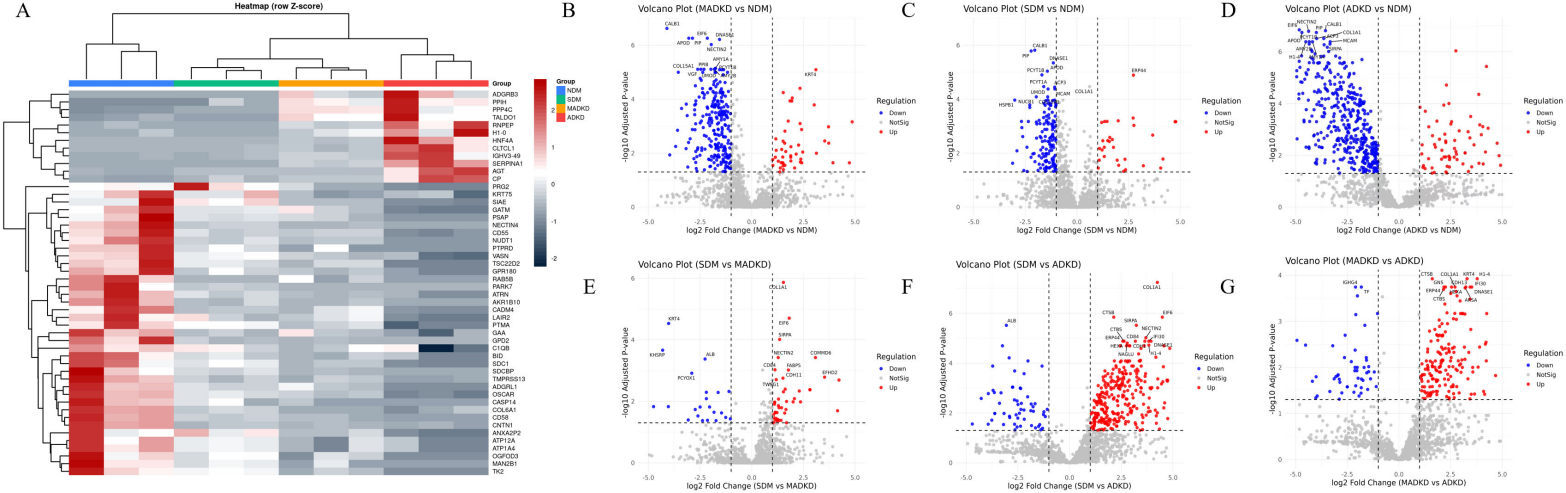
Differential Proteomics Analysis. **(A)** Heatmap of the Top 50 Proteins. **(B)** Volcano Plot of Group MADKD versus NDM. **(C)** Volcano Plot of Group SDM versus NDM. **(D)** Volcano Plot of Group ADKD versus NDM. **(E)** Volcano Plot of Group SDM versus MADKD. **(F)** Volcano Plot of Group SDM versus ADKD. **(G)** Volcano Plot of Group MADKD versus ADKD.

The expression matrix was extracted from the raw data. When the proportion of the minimum positive value exceeded 2%, all values approximating this threshold were set to N/A. Additionally, zeros were uniformly treated as non-detected values and replaced with N/A. The detection rate—defined as the proportion of non-missing values across all samples for each protein (row)—was then calculated. Only proteins with a detection rate strictly greater than 50% were retained for subsequent analysis. Based on the preprocessed protein expression data, a multidimensional analytical strategy was employed to systematically identify key biomarkers. First, we performed multi-group differential analysis using the Limma package, which included an overall F-test and pairwise group comparisons. Concurrently, the kurtosis of expression distributions was calculated to assess data concentration, and Spearman correlation analysis was applied to quantify the monotonic trend of protein expression along disease progression. We established three combined screening criteria: global significance (F-FDR<0.05), high kurtosis (kurtosis>3), and a strong monotonic trend (|Spearman’s correlation coefficient|>0.8). This approach efficiently identified key proteins that were statistically significant, exhibited well-defined distribution characteristics, and were closely associated with disease progression (as shown in **Figure 4**), thereby providing high-quality candidate targets for further investigation.

The heatmap in **Figure 4A** clearly illustrates the expression patterns of 50 key proteins across different stages of diabetic kidney disease (NDM→SDM→MADKD→ADKD). Following row-wise Z-score normalization, it is evident that the majority of proteins exhibit pronounced trends in expression levels along with disease progression. Notably, approximately two-thirds of the proteins were significantly downregulated in the ADKD group.

Volcano plots comparing each diabetic stage to the healthy control group (**Figures 4B–D**) revealed the top five most significantly altered proteins. In the ADKD vs. NDM comparison, these were eukaryotic translation initiation factor 6 (EIF6), apolipoprotein D (APOD), nectin cell adhesion molecule 2 (NECTIN2), prolactin induced protein (PIP), and calbindin 1 (CALB1). For MADKD vs. NDM, the top proteins were CALB1, PIP, deoxyribonuclease 1 (DNASE1), APOD, and phosphate cytidylyltransferase 1B, choline (PCYT1B). In the SDM vs. NDM comparison, the most significantly changed proteins were CALB1, PIP, APOD, EIF6, and DNASE1. Notably, all these proteins were downregulated.

The volcano plots (**Figures 4E–G**) reveal the top five most significantly altered proteins at different stages of Diabetic Kidney Disease progression. For ADKD vs. SDM, these proteins are collagen type I alpha 1 chain (COL1A1), albumin (ALB), cathepsin B (CTSB), EIF6, and signal regulatory protein alpha (SIRPA). For MADKD vs. SDM, the significant proteins are COL1A1, EIF6, keratin 4 (KRT4), SIRPA, and KH-type splicing regulatory protein (KHSRP). For ADKD vs. MADKD, they are CTSB, KRT4, H1.4 linker histone, cluster member (H1-4), immunoglobulin heavy constant gamma 4 (IGHG4), and transferrin (TF). These distinct protein combinations, which change specifically at different stages, suggest their potential as biomarkers for distinguishing between the various progression phases of Diabetic Kidney Disease.

#### 3.3.3 Screening of marker proteins

**Figure 5:**
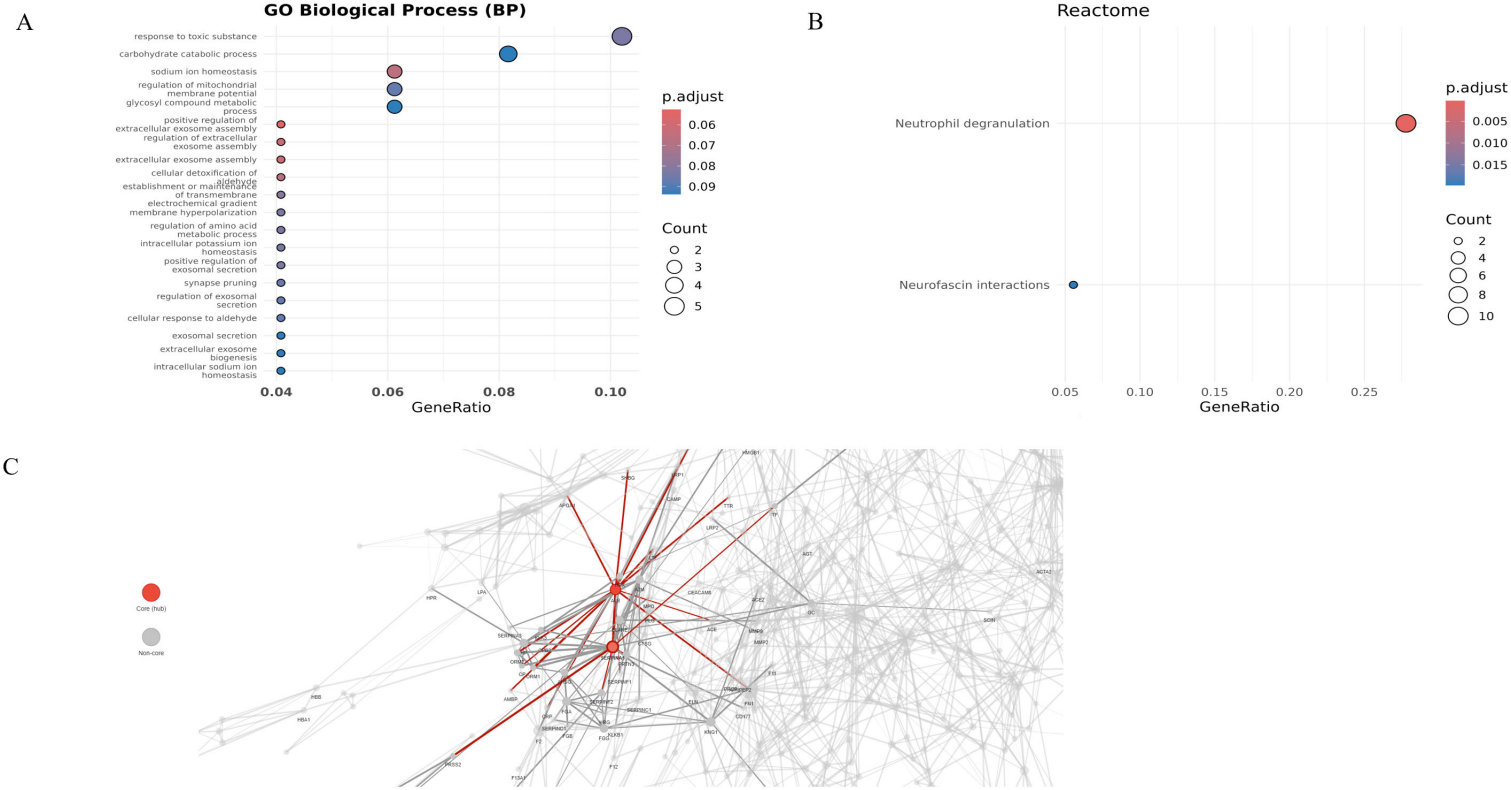
Protein Functional Analysis and Candidate Protein Screening for Validation. **(A)** GO Enrichment Analysis. **(B)** Reactome Pathway Analysis. **(C)** Protein-Protein Interaction (PPI) Network.

To systematically elucidate the biological functions of the differentially expressed proteins, we performed Gene Ontology (GO) biological process and Reactome pathway enrichment analyses on the screened differential proteins (FDR<0.05) using the clusterProfiler software package, setting an FDR<0.05 as the significant enrichment threshold (as shown in **Figure 5**).

The functional enrichment analysis based on the differentially expressed proteins revealed (see **Figure 5A**) that the pathogenesis and progression of Diabetic Kidney Disease (DKD) are closely associated with disruptions across multiple biological levels. GO biological process annotation analysis indicated that the significantly differentially expressed proteins were primarily involved in several processes: response to toxic substance — including collagen type VI alpha 1 chain (COL6A1), ceruloplasmin (CP), syndecan 1 (SDC1), Parkinsonism associated deglycase (PARK7), and aldo−keto reductase family 1 member B10 (AKR1B10); carbohydrate catabolic process — involving COL6A1, alpha glucosidase (GAA), glycerol−3−phosphate dehydrogenase 2 (GPD2), and mannosidase alpha class 2B member 1 (MAN2B1); sodium ion homeostasis — mediated by angiotensinogen (AGT), ATPase H+/K+ transporting non−gastric alpha2 subunit (ATP12A), and ATPase Na+/K+ transporting subunit alpha 4 (ATP1A4); regulation of mitochondrial membrane potential — associated with BH3 interacting domain death agonist (BID), COL6A1, and PARK7; and glycosyl compound metabolic process — involving nudix hydrolase 1 (NUDT1), protein tyrosine kinase 2 (TK2), and AKR1B10.

Reactome pathway analysis further revealed specific pathway alterations from the perspective of molecular interaction networks (see **Figure 5B**). The most significantly enriched pathways included Neutrophil degranulation and Neurofascin interactions. The significant enrichment of the Neutrophil degranulation pathway suggests that innate immune responses and inflammatory reactions may play a key role in DKD progression, which aligns with the clinically observed infiltration of inflammatory cells in the kidney (12). The enrichment of the Neurofascin interactions pathway implies that abnormalities in intercellular interactions and connections may contribute to disease pathogenesis, providing a new perspective for understanding impaired cell-cell communication in DKD.

These analytical results collectively construct a multi-layered disease mechanism network: from fundamental ion homeostasis imbalance and protein metabolic disturbances, to oxidative stress and alterations in the extracellular microenvironment, and further to immune-inflammatory responses and dysregulation of cell junctions. Abnormalities in biological processes across these various layers together constitute the complex pathophysiological characteristics of DKD (12).

Through GO and Reactome pathway analyses, 25 core proteins were screened: CNTN1, syndecan binding protein (SDCBP), CD58, CD55 molecule (CD55), GAA, MAN2B1, SERPINA1, proteoglycan 2 (PRG2), prosaposin (PSAP), member RAS oncogene family (RAB5B), osteoclast associated Ig-like receptor (OSCAR), COL6A1, CP, SDC1, PARK7, AKR1B10, GAA, GPD2, MAN2B1, AGT, ATP12A, ATP1A4, BID, NUDT1, TK2.

To systematically understand the functional associations among proteins within the context of this study, a Protein-Protein Interaction (PPI) network was constructed based on the expression profiles of these 25 differentially expressed proteins. The analysis results showed that SERPINA1 (Alpha-1-antitrypsin) exhibited significant centrality across all evaluated metrics and was identified as one of the most critical hub nodes in the entire network (see **Figure 5C**). This indicates that SERPINA1 plays an indispensable “bridging” role in controlling information flow and facilitating communication between functional modules within the network.

### 3.4 Validation by ELISA

**Figure 6:**
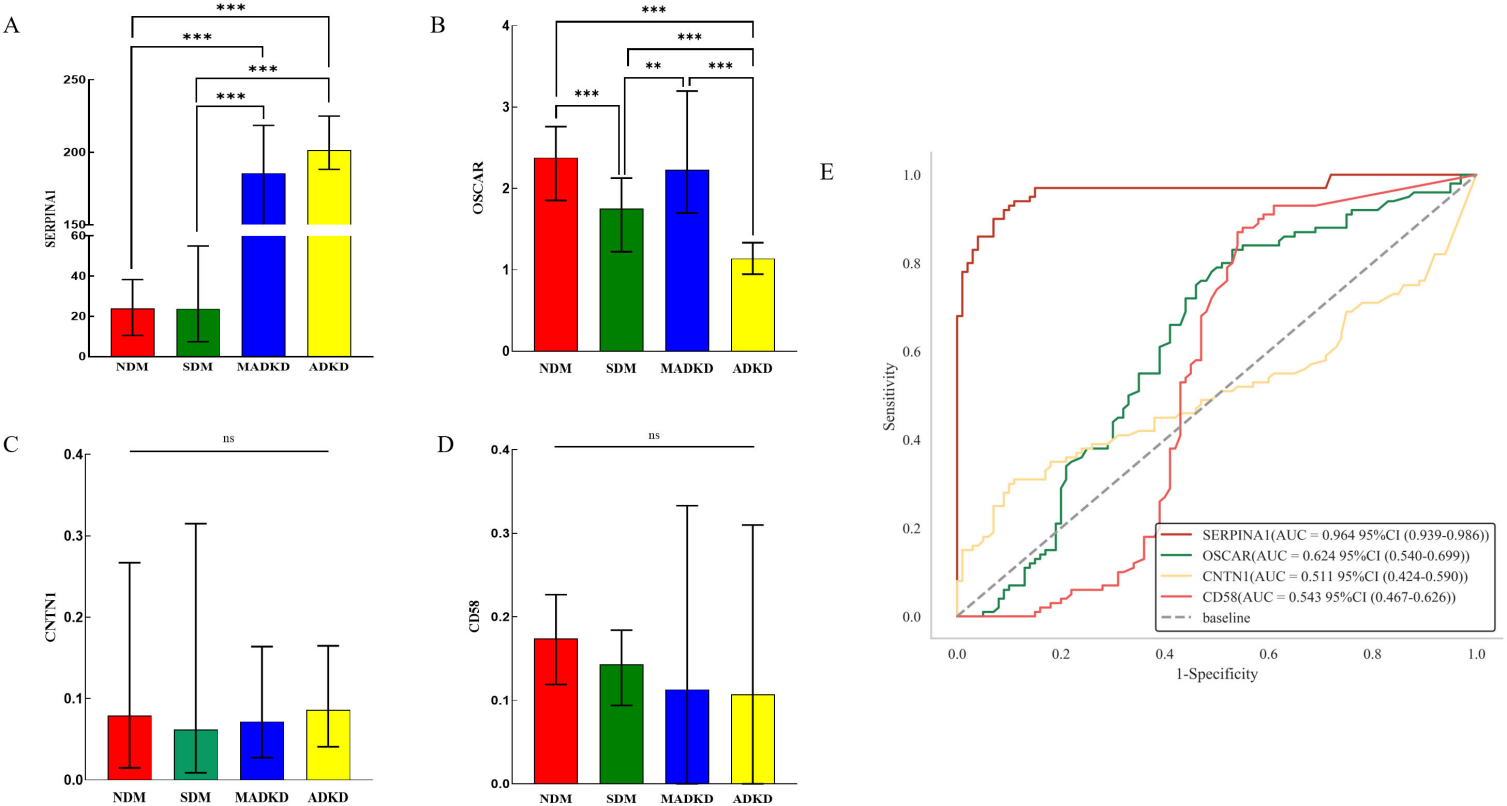
The OD change values of the four screened proteins in ELISA reactions. **(A)** Changes in OD values of SERPINA1; **(B)** Changes in OD values of OSCAR; **(C)** Changes in OD values of CNTN1; **(D)** Changes in OD values of CD58; **(E)** ROC Curves of the Four Screened Proteins. The overall difference in SERPINA1 and OSCAR levels was significant; pairwise comparisons without annotation indicated no statistical significance (ns). The overall difference in CNTN1and CD58 was not statistically sianificant (ns). *P<0.05, ** *P*<0.01, *** *P*<0.001.

To validate the screening results, enzyme-linked immunosorbent assays (ELISA) were performed to quantify the levels of four candidate proteins (SERPINA1, OSCAR, CNTN1, CD58) across all 250 samples, which included healthy controls and diabetic patients at various disease stages. The relative concentrations, expressed as optical density (OD) values, are detailed in **Figures 6A–D**.

The results revealed highly significant alterations in SERPINA1 concentration across groups (*P* < 0.001). Although its levels were comparable between the healthy control (NDM) and normoalbuminuric (SDM) groups, pairwise comparisons between the diabetic kidney disease groups (MADKD and ADKD) and the non−diabetic kidney disease groups (NDM and SDM) all showed significant differences, strongly suggesting that SERPINA1 may indicate the early onset of diabetic kidney disease and play a key role in the pathogenesis of DKD. The lack of difference between ADKD and MADKD likely reflects the ELISA’s upper detection limit being reached in ADKD samples, as further dilution reduced precision. OSCAR levels also differed significantly among groups (*P* < 0.001), but exhibited a distinct pattern, decreasing to its lowest level in the ADKD group, while no significant difference was observed between the MADKD group and the NDM group, indicating its insensitivity in diagnosing DKD and its complex regulatory dynamics.

In contrast, the OD signals for CNTN1 and CD58 were consistently detected at very low levels across all groups, with no statistically significant inter-group differences observed (*P* = 0.879 and *P* = 0.230, respectively). We posit that this likely reflects a methodological limitation wherein the actual protein concentrations fell below the quantitative detection limit of the ELISA kits employed, resulting in OD readings near background levels that precluded reliable quantification.

Further using the urinary albumin-to-creatinine ratio as the practical clinical reference, the diagnostic efficacy of these four proteins was evaluated via receiver operating characteristic (ROC) curves (**Figure 6E**). SERPINA1 demonstrated outstanding, near-perfect diagnostic performance, with an area under the curve (AUC) of 0.964 (95% CI: 0.936–0.990). In contrast, the diagnostic performance of the other three proteins was poor: OSCAR had an AUC of 0.624 (95% CI: 0.539–0.684), CNTN1 an AUC of 0.511 (95% CI: 0.416–0.586), and CD58 an AUC of 0.543 (95% CI: 0.450–0.636).

In summary, SERPINA1 exhibits extremely high diagnostic potential based on ACR, whereas OSCAR, CNTN1, and CD58 show limited value as standalone biomarkers. These findings underscore the promise of SERPINA1 for clinical application and warrant its further validation.

## 4 Discussion

Epidemiological data indicate that diabetes and hypertension are the leading causes of chronic kidney disease (CKD). A 2019 survey reported that over 80% of CKD cases are attributable to these two conditions (13). The global prevalence of DKD is rising rapidly, paralleling the increasing rates of diabetes and hypertension. However, effective therapeutic options to slow DKD progression remain limited, a challenge compounded by difficulties in early diagnosis. Although eGFR and ACR are standard biomarkers for renal function assessment and can identify some DKD patients, they lack sufficient sensitivity and specificity. Consistent with this notion, our study also found that eGFR has limited utility in detecting minor kidney damage in diabetic patients. Consequently, DKD often goes undetected until significant renal impairment has occurred.

Among traditional urinary protein biomarkers, UTRF has demonstrated high sensitivity and specificity in diagnosing diabetic kidney disease. Previous literature has also reported that urinary excretion of transferrin in T2DM patients can effectively predict the onset of diabetic kidney disease (14). Another study suggested that this is because transferrin accumulates in the cytoplasm of glomerular podocytes during the early stages of DKD (15). Research has also observed that urinary transferrin is associated with progressive changes, such as interstitial fibrosis, atrophic tubular cells, and inflammatory cell infiltration in the Renal Interstitium (16). Therefore, dysregulation of iron homeostasis is closely linked to the development of DKD (17).

Secondly, UIgG has also shown excellent diagnostic performance, consistent with previous reports that urinary IgG levels can predict the occurrence of microalbuminuria (14). Several studies have reported that Uα1-MG, Uβ2-MG, and URBP can predict early renal injury in diabetes (18). However, this study found that the diagnostic efficacy of Uα1-MG, Uβ2-MG, and URBP in diagnosing diabetic kidney disease is inferior to that of UTRF and UIgG.

Based on reports linking urinary amino acids to the progression of DKD (19), this study further investigated the potential of 20 urinary amino acids as diagnostic biomarkers for DKD. This research found that urinary amino acids undergo changes as early as the diabetes stage, indicating that alterations in amino acid metabolism could be used for diagnosing prediabetes and type 2 diabetes. This aligns with existing studies (20, 21). Regarding predicting the onset of DKD in the diabetes stage, urinary amino acids demonstrated limited diagnostic efficacy. Only proline (PRO) showed predictive value for advanced DKD progression (AUC = 0.746, sensitivity 77%, specificity 59%). This finding is consistent with a 2023 study (22) which reported elevated proline levels in the urine of DKD patients, collectively suggesting that PRO may serve as a potential biomarker for advanced DKD progression. Notably, our study innovatively found that the combined diagnostic model of all 20 amino acids achieved high diagnostic value, even outperforming traditional urinary protein markers. Although individual urinary amino acids may be susceptible to various confounding factors, their combination can mitigate these influences and enhance diagnostic performance.

Although some literature has indicated that citrulline (Cit) is associated with the development and progression of DKD (23), that aspartic acid (Asp) is significantly elevated in DKD patients (22), and that low urinary tyrosine levels are linked to the occurrence of DKD (24), the aforementioned amino acids did not show significant differences between the MADKD and the SDM in this study cohort, and their overall diagnostic efficacy was limited.

This discrepancy may stem from the following reasons: population characteristics and dietary structure could be significant influencing factors; disease heterogeneity and comorbidities – DKD patients often have comorbidities such as hypertension and hyperuricemia, and the corresponding medications for these conditions may independently affect amino acid metabolic pathways, thereby diminishing their efficacy as specific biomarkers.

This study, based on the evaluation of the diagnostic efficacy of traditional urinary protein biomarkers and urinary amino acid biomarkers, innovatively compared the diagnostic performance of these two categories of biomarkers in diagnosing diabetic kidney disease. Our findings revealed that urinary amino acids demonstrated inferior diagnostic performance compared to traditional urinary protein biomarkers. Among these, UTRF and UIgG exhibited the best diagnostic efficacy within their respective categories.

We believe that an ideal biomarker should dynamically change with disease progression, not only aiding in diagnosing disease onset but also useful for assessing disease prognosis and progression stages. Therefore, in the proteomic analysis of this study, we focused on differentially expressed proteins that showed regular changes with disease progression. Among these, SERPINA1 was identified as a key protein in this changing process.

SERPINA1 (α1-antitrypsin) is a serine protease inhibitor primarily synthesized by the liver and belongs to the Serpin superfamily. It plays a crucial antiprotease role in the systemic circulation, with its main function being the inhibition of neutrophil elastase, thereby protecting tissues from damage caused by excessive inflammatory responses. It is widely reported in the literature that SERPINA1 gene deficiency leading to α1-antitrypsin deficiency is closely associated with the occurrence of lung diseases (such as COPD) (25, 26) and liver diseases (such as cirrhosis) (27). Furthermore, changes in its expression levels are also considered to be related to the progression of various inflammatory and fibrotic diseases (28). However, there is currently no clear report on the specific expression pattern of SERPINA1 in diabetic kidney disease or its potential value as a diagnostic biomarker for this disease, and its specific role in the pathogenesis of diabetic kidney disease still requires further investigation. While ACR-guided stratification is clinically accepted for DKD staging, future studies should validate SERPINA1 against eGFR trajectories or histopathological criteria where feasible.

Although SERPINA1 demonstrates great potential as a diagnostic biomarker for DKD in this study, it must be acknowledged that its expression is regulated by multiple factors. Notably, as an acute-phase protein, SERPINA1 levels can rise significantly under infectious and inflammatory conditions, and its concentration is positively correlated with disease severity (29). Additionally, studies have also reported that COVID-19 infection can increase serum SERPINA1 levels (30). These reports indicate that inflammation can elevate serum SERPINA1 levels, which may subsequently lead to increased urinary SERPINA1 excretion, thereby constituting a potential confounding variable that cannot be ignored in DKD-specific diagnosis. At the same time, SERPINA1’s utility in DKD-specific diagnosis warrants validation in cohorts with competing renal pathologies, though its strong association with albuminuria progression here supports initial clinical relevance.

Through systematic screening and validation, the 25 key proteins obtained in this study further reveal, at the molecular level, the close association between diabetic kidney disease and several key biological processes, including response to toxic substances, carbohydrate catabolism, sodium ion homeostasis, regulation of mitochondrial membrane potential, and glycosyl compound metabolism. These processes highly align with the core pathological mechanisms of diabetic kidney disease widely reported in previous studies—including metabolic disorders, oxidative stress, ion homeostasis imbalance, and mitochondrial dysfunction (31–33). The findings of this study not only confirm the important role of the aforementioned mechanisms in disease progression from a proteomics perspective but also suggest that these key proteins may collectively drive the occurrence and development of diabetic kidney disease by regulating related pathways, providing new molecular clues and potential intervention targets for subsequent mechanistic exploration.

## 5 Conclusions

This study found that traditional urinary protein markers, such as UTRF and UIgG, demonstrated strong and robust performance in diagnosing DKD, with their diagnostic efficacy (AUC>0.9) significantly superior to that of urinary amino acid markers, further consolidating their clinical value. Although the urinary amino acid profile showed alterations in the early stages of diabetes, its diagnostic value was limited and more likely reflected systemic metabolic disturbances rather than kidney-specific injury. When comparing individual markers, the diagnostic efficiency of single urinary amino acids was lower than that of traditional urinary proteins. However, when all 20 urinary amino acids were combined for diagnosis, the combined model exhibited excellent diagnostic performance (AUC = 0.928). The most important finding of this study was the identification and validation of the urinary protein SERPINA1 (alpha-1-antitrypsin) as an excellent novel diagnostic biomarker through proteomic screening. Its expression was significantly upregulated with disease progression, and it demonstrated exceptionally high discriminatory power (AUC = 0.964) when validated by ELISA in a large cohort, indicating substantial potential for clinical application. In summary, this study not only confirmed the reliability of traditional markers but, more importantly, discovered the highly promising next-generation biomarker SERPINA1, laying a molecular foundation for the development of high-precision, non-invasive diagnostic strategies for DKD. Future large-scale prospective cohort studies are needed to advance its clinical translation.

## Data Availability

The original contributions presented in the study are included in the article/supplementary material. Further inquiries can be directed to the corresponding authors.
